# A case of generalized pustular psoriasis following Moderna/NIAID COVID-19 vaccination successfully treated with secukinumab^☆^

**DOI:** 10.1016/j.abd.2023.07.016

**Published:** 2024-06-26

**Authors:** Misaki Kusano, Ryuto Mukaiyama, Toshiyuki Yamamoto

**Affiliations:** Department of Dermatology, Fukushima Medical University, Fukushima, Japan

Dear Editor,

Here, we present a case of the development of generalized pustular psoriasis (GPP) following COVID-19 vaccination in a patient with psoriasis vulgaris.

A 64-year-old woman was referred to our department, complaining of high fever, and erythemas with superficial pustules all over the body seven days after the third dose of Moderna/NIAID COVID-19 messenger RNA vaccination (mRNA-CV). She had received the first and second doses of Pfizer/BioNTech mRNA-CV. She had developed psoriasis vulgaris (PsV) about one and half years previously and had been treated with oral apremilast and topical calcipotriol hydrate at a nearby clinic under control. At the initial visit, a physical examination revealed multiple erythemas and small pustules with scales on the trunk and extremities, and edema was prominent on the bilateral lower legs (Fig. 1). A skin biopsy was performed from an erythema with pustules on the left thigh. Histopathology showed subcorneal aggregation of neutrophils surrounded by multilocular small pustules with spongiosis (Kogoj’s spongiform pustule) (Fig. 2). Blood test showed that C-reactive protein is high (7.80 mg/dL) and white blood cells were normal. Liver and kidney dysfunction was not observed. The patient was treated with secukinumab, resulting in a significant improvement of the rash after 2 months.

**Fig. 1:**
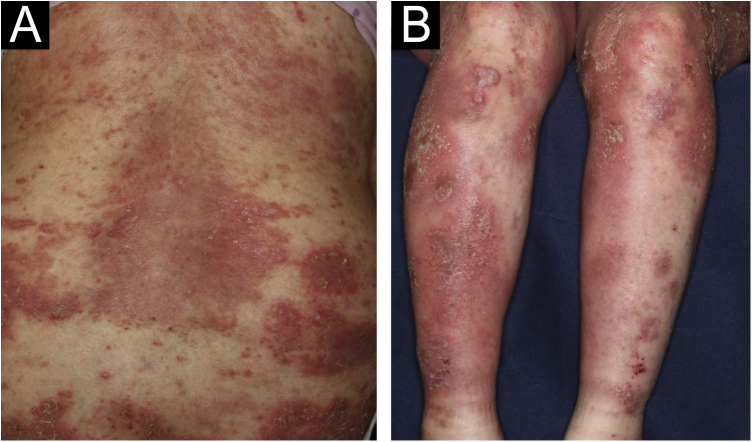
(A and B) Multiple erythemas and small pustules with scales on the trunk and extremities, and edema was prominent on both lower legs.

**Fig. 2:**
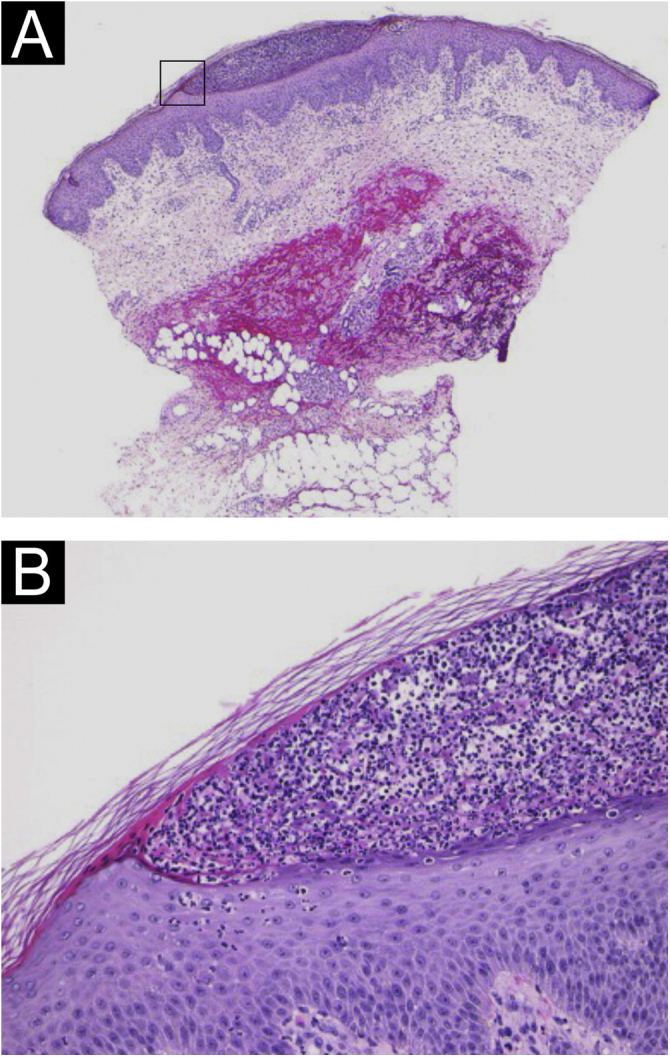
(A) Subcorneal pustule formation (Hematoxylin & eosin, ×20). (B) Subcorneal aggregation of neutrophils surrounded by multilocular small pustules with spongiosis (Kogoj’s spongiform pustule, ×200).

In recent years, there have been several reports of GPP following the mRNA-CV.^1^, ^2^, ^3^, ^4^, ^5^, ^6^, ^7^, ^8^ As far as we searched, nine cases have been reported (Table 1). The average age was 52 years and the male-to-female ratio was 5:4. Seven of the nine patients had a history of psoriasis, whereas two patients developed GPP de novo. The duration from vaccination to onset of symptoms ranged from 4 to 21 days. mRNA vaccines were used in 7 of 9 cases (5 cases were Pfizer). Most patients developed pustular lesions after the first or second vaccination. Acitretin (n = 3), etretinate (n = 1), cyclosporine (n = 1), and biologics (n = 6) were used for treatment (overlapping). Regarding the biologics, secukinumab (n = 3), infliximab (n = 1), adalimumab (n = 1), and risankizumab (n = 1) were used. Two of the nine patients performed a sequencing analysis of genomic DNA derived from peripheral blood, which revealed no gene mutations in entire coding regions of IL36RN.^8^ In our case, IL36RN was not investigated.

**Table 1:** Cases of generalized pustular psoriasis following mRNA coronavirus vaccination.

Author	Age	Sex	History	Vaccine	Dose	Days of onset	Treatment	IL-36RN
Onsun N	72	Male	Psoriasis vulgaris	Inactivated vaccine	1st	4	acitretin,infliximab	–
Elamin S	66	Female	None	DNA(Oxford-AstraZeneca)	1st	21	acitretin	–
Perna D	40	Male	Psoriasis vulgaris	ｍRNA	1st	5	secukinumab	–
Yamazaki K (in Japanese)	76	Male	Psoriasis vulgaris	Pfizer/BioNTech BNT162b2 mRNA	2nd	19	adalimumab	–
Yatsuzuka K	65	Male	Generalized pustular psoriasis	ｍRNA	2nd	12	secukinumab	–
Frioui R	20	Male	Psoriasis vulgaris	Pfizer/BioNTech BNT162b2 mRNA	1st	4	acitretin	–
Pavia G	47	Female	Psoriasis vulgaris	Pfizer/BioNTech BNT162b2 mRNA	2nd	10	risankizumab	–
Tachibana K	60	Female	Impetigo herpetiformis	Pfizer/BioNTech BNT162b2 mRNA	2nd	8	etretinate	none
Tachibana K	18	Female	Psoriasis vulgaris	Pfizer/BioNTech BNT162b2 mRNA	1st	7	cyclosporine, secukinumab	none
This case	64	Female	Psoriasis vulgaris	Moderna/NIAID ｍRNA-1273	3rd	7	secukinumab	–

Several mechanisms have been proposed to exacerbate psoriasis by COVID-19 vaccination. One is the mechanism involving angiotensin converting enzyme (ACE). It is believed that the activity of ACE increases and causes an inflammatory cytokine storm by COVID-19 vaccination.^9^ The second is the activation of the toll-like receptor (TLR) pathway. Farkas et al.^10^ found that vaccines may activate dermal myeloid dendritic cells that play roles in the inflammatory psoriasis cascade. Dendritic cells differentiate T-cells into Th1 and Th17 cells by releasing inflammatory mediators and then trigger the release of downstream cytokines. These inflammatory cytokines are thought to be involved in the exacerbation of psoriasis,^1^ but further examination is necessary to clarify the mechanisms of GPP induction by COVID-19 vaccination.

## Financial support

None declared.

## Authors’ contributions

Misaki Kusano: Approval of the final version of the manuscript; critical literature review; data collection; analysis and interpretation; study conception and planning; management of studied cases; manuscript critical review; preparation and writing of the manuscript.

Ryuto Mukaiyama: Approval of the final version of the manuscript; critical literature review; manuscript critical review; preparation and writing of the manuscript.

Toshiyuki Yamamoto: Approval of the final version of the manuscript; critical literature review; data collection; analysis and interpretation; study conception and planning; manuscript critical review; preparation and writing of the manuscript.

## Conflicts of interest

None declared.
